# Solid Dispersion of Hesperidin Alleviates Acetic Acid-Induced Colitis Through Modulating the Gut Microbiota Dysbiosis in Rats

**DOI:** 10.3390/foods14183252

**Published:** 2025-09-19

**Authors:** Qiru Wang, Dan Liu, Qi Wu, Yanling Sun, Ning Ma, Xin He, Xinghua Zhao

**Affiliations:** College of Veterinary Medicine, Hebei Agricultural University, Baoding 071001, China; 15635403877@163.com (Q.W.); 13273213194@163.com (D.L.); wq319984068@163.com (Q.W.); 18713227716@163.com (Y.S.); maning9618@163.com (N.M.)

**Keywords:** hesperidin, solid dispersion, functional food, colitis, inflammation, gut microbiota

## Abstract

Hesperidin (HD) is predominantly found in citrus fruits, and has been shown to possess various biological properties, such as anti-inflammatory, antioxidant, and anti-carcinogenic. However, its application is limited by poor solubility. In this study, a new solid dispersion (SD) of hesperidin was prepared by ball milling using PVPK30 as the carrier, and the in vitro and in vivo studies and the therapeutic effects about colitis in rats were evaluated. In vitro analysis revealed that the solid dispersion showed a better release effect. The cumulative release of HD-SD reached 48.24% at 120 min, which was 5.9 times that of pure HD. In vivo studies demonstrated that C_max_ and AUC_0__–__24_ were significantly higher in HD-SD compared with pure HD (*p* < 0.01), which were 2.67 and 1.50 times that of HD, respectively. Furthermore, treatment with HD-SD significantly alleviates symptoms and histological features in acetic acid (AA)-induced colitis in rats. Furthermore, HD-SD treatment significantly ameliorated the disease severity of acetic acid (AA)-induced colitis in rats, as evidenced by improved clinical signs, attenuated histological damage, and decreased levels of inflammatory factors (TNF-α, IL-6, and IL-1β). Moreover, the structure and relative abundance of the gut microbiota were modulated. Specifically, the relative abundance of *Erysipelotrichaceae* was decreased and the relative abundance of *Bacteroidota*, *Lachnospiraceae*, and *[Eubacterium]_coprostanoligenes_group* were increased. These results suggest that HD-SD could serve as a gut-health-promoting functional ingredient, potentially contributing to the dietary management of colitis through microbiota modulation.

## 1. Introduction

Inflammatory bowel disease (IBD) is a chronic inflammatory disorder in the gastrointestinal system which includes Crohn’s disease (CD) and ulcerative colitis (UC) [1]. The rates of incidence and prevalence of UC in Western Europe and North America are greater than those found in Asia. However, in recent years, there has been a rising trend in the incidence of UC in Asian populations [2]. The most common symptoms of UC include abdominal pain, diarrhea, mucus and blood in the stool, and weight loss. While the exact etiology of UC remains unclear, growing evidence supports the notion that poor dietary patterns and gut microbial dysbiosis significantly increase the risk of developing UC [3,4]. Increased secretion of inflammatory factors in the intestinal mucosa and dysbiosis in the gut microbiota play significant roles in the pathogenesis of UC [5]. Recent studies have indicated that flavonoids exhibit promising effects in preventing and ameliorating UC [6]. Additionally, natural active compounds are advantageous due to their low toxicity, minimal side effects, and affordability, making them potential candidates for further exploration as preventive or therapeutic agents for chronic UC.

Fortunately, plant-derived functional components from dietary sources have emerged as promising candidates for the prevention and management of colonic disorders. Researchers in food science and nutrition are actively investigating how bioactive compounds sourced from food can modulate intestinal inflammation and maintain microbial homeostasis, providing innovative strategies for the intervention of UC [7,8]. Hesperidin (HD) is a natural flavonoid compound found in the *Rutaceae* family, primarily extracted from *citrus* fruits such as oranges, sweet oranges, and lemons, and it is the most abundant component in sweet orange juice, with a concentration of 28.6 mg/100 mL [9,10]. Hesperidin can be used as a dietary supplement to treat obesity, atherosclerosis, and promote cardiovascular health [11,12]. In addition, HD possesses a range of pharmacological effects, including antibacterial [13], antioxidant [14], anti-inflammatory [15], the modulation of gut microbiota [16], and anticancer [17]. Earlier studies have shown that supplementation with HD can upregulate the Nrf2 antioxidant pathway in mice and reduce the levels of inflammatory cytokines, thereby improving the symptoms of colitis [18]. Previous studies have indicated that 200 mg/kg hesperidin in rats regulated the gut microbiota by increasing the total bacterial count, particularly increasing the proportion of *Lactobacillus* and *Bifidobacterium* [19]. Despite numerous studies that demonstrated that HD had broad application prospects as a new additive and therapeutic agent in the pharmaceutical and food industries, its poor water solubility (only 4.95 μg/mL) led to low bioavailability, which limited its applications in clinical medicine and functional food field [20,21]. In recent years, various formulations of HD, such as nanoparticles [22], nanosuspensions [23], and inclusion complexes [24] have been utilized to address its poor aqueous solubility and low oral bioavailability.

Solid dispersions facilitate the transformation of the active pharmaceutical ingredient (API) from a crystalline state to a molecular, microcrystalline, or amorphous state, which enhances the solubility and bioavailability of the API [25]. The SD technology can enhance the solubility of poorly plant-derived functional components and improve oral bioavailability, which has been widely applied in the functional food field [26]. Common methods for preparing SD include solvent evaporation, spray drying, hot-melt extrusion, and ball milling. This study employed the environmentally friendly and cost-effective ball milling method with PVPK30 as the carrier to prepare the hesperidin solid dispersion (HD-SD). The physicochemical properties, solubility, and pharmacokinetic behaviors were evaluated. Furthermore, this study evaluated the therapeutic effects of HD-SD on acetic acid-induced colitis in rats.

## 2. Methods and Materials

### 2.1. Materials

HD (purity ≥ 95%) was provided by Shanghai yuanye Bio-Technology Co., Ltd. (Shanghai, China). HD standard (purity ≥ 98%) was obtained from Beijing Vokai Biotechnology Co., Ltd. (Beijing, China). PVPK30 was purchased from Aladdin Biochemical Technology Co., Ltd. (Shanghai, China). Chromatographic grade methanol was purchased from Thermo Fisher Scientific (Shanghai, China). Carboxymethylcellulose sodium (CMC-Na) was purchased from Beijing Solarbio Science & Technology Co., Ltd. (Beijing, China). Acetic acid (AA) was purchased from Fuyu Chemical Co., Ltd. (Tianjin, China).

### 2.2. Preparation of HD Solid Dispersion by Ball Milling Method

HD and PVPK30 were mixed in different weight ratios (1:9, 2:8, 3:7, 4:6, 5:5) in 50 mL centrifuge tubes using a vortex mixer (VORTEX-5, Kylin-Bell, Haimen, China) for 3 min to obtain the physical mixture (named HD-PM). Appropriate amounts of the physical mixture were transferred into 2 mL centrifuge tubes containing one zirconium oxide ball (3 mm diameter). Then, the physical mixture was milled at 1200 rpm using a micro ball mill GT300 (Grinder, Beijing, China) at room temperature (22–25 °C). The milling time was 30 min. After the completion of the milling treatment, the obtained systems were named HD-SD.

### 2.3. The Characterization of HD Solid Dispersions

#### 2.3.1. Powder X-Ray Diffraction (PXRD)

PXRD of HD, PVPK30, and different weight ratios of HD-SD and HD-PM were obtained at room temperature using a D8 Advance PXRD (TD-3700, Dandong Tongda Science & Technology Co., Ltd., Liaoning, China). Measurements were conducted using Cu Kα radiation (λ = 1.54178 Å) with the X-ray tube operating at 45 kV and 40 mA. A scanning range of 3° to 40° (2θ) was applied with a step size of 0.015° and a dwell time of 0.1 s per step.

#### 2.3.2. Differential Scanning Calorimetry (DSC)

HD, PVPK 30, HD-SD (1:9), and HD-PM (1:9) were set in standard aluminum pans for DSC analysis using a Mettler Toledo instrument (Zurich, Switzerland). The samples were heated from 25 to 300 °C at a scanning rate of 10 °C/min under a nitrogen flow rate of 50 mL/min.

#### 2.3.3. Fourier Transform Infrared Spectroscopy (FTIR)

FT-IR spectra of HD, PVPK30, HD-PM (1:9), and HD-SD (1:9) samples were recorded using a Bruker ALPHA spectrometer (Bruker GmbH, Ettlingen, Germany). Each sample was mixed with KBr powder in a 1:100 ratio. The resulting powder was then pressed into pellets for scanning, with a spectral range of 4000 to 400 cm^−1^ and a resolution of 0.1 cm^−1^.

### 2.4. Study on Dissolution In Vitro

The dissolution study of HD, HD-PM (1:9), and HD-SD (1:9) (containing 36 mg HD) was studied by the paddle method with a dissolution apparatus (RC-8DS, Tianjin Tuopu Instrument Co., Ltd., Tianjin, China) [27]. The studies were carried out at 37 ± 0.5 °C with a stirring speed of 100 rpm in 900 mL of dissolution medium (pH 6.8). The powders were sieved through 100-mesh sieves (150–180 μm). At time intervals of 5, 10, 15, 30, 45, 60, 90, and 120 min, 1 mL of sample was collected, and equivalent volumes of fresh dissolution medium were used for replacement. Each sample was then filtered with a 0.22 μm syringe-driven filter. The samples were examined using UV/vis spectrophotometry (UV-6850, Jenway, Chicago, IL, USA) at 284 nm to avoid interference from PVPK30. The calculation of the cumulative release rate was based on the formula described by Gao et al. [28].

### 2.5. Stability Analysis

An appropriate amount of HD-SD (1:9) was placed into a humidity chamber (Shanghai Yiheng Scientific Instruments Co., Ltd., Shanghai, China) with 40 ± 0.5 °C and 75 ± 5% RH for 180 d. Then, PXRD analysis was carried out at 0, 30, 90, 120, 150, and 180 days to assess any potential crystallization of solid dispersions.

### 2.6. In Vivo Pharmacokinetic Assay

All experiments were approved by the Ethics Committee of Experimental Animal Care of Hebei Agricultural University (No. 2021058). Twelve male Sprague–Dawley (SD) rats (220 ± 20 g) were purchased from SPF Biotechnology Co., Ltd. (Beijing, China). After 1 week of adaptation, the rats were randomly divided into the HD group and the HD-SD (1:9) group (*n* = 6 per group). The rats of the two groups received an oral administration of 100 mg/kg HD (or equivalent to the amount of HD) suspended in a 0.5% CMC-Na aqueous solution, respectively. Blood samples (approximately 500 μL) were withdrawn from the orbital sinus at 0.083, 0.167, 0.25, 0.5, 0.75, 1, 1.5, 2, 4, 6, 8, 10, 12, and 24 h after oral administration and placed in heparinized Eppendorf tubes. Blood samples were centrifuged at 6000× *g* for 10 min and the plasma was separated and stored at −80 °C until analysis.

Precisely, 100 μL of plasma sample was taken and mixed with 300 μL of methanol, vortexed for 3 min, then centrifuged at 12,000× *g* for 10 min. The supernatant was taken out, dried by nitrogen gas, and the residue was reconstituted in 150 µL methanol, and then injected for HPLC (Waters 1525 series, Waters Corporation, MA, USA) analysis. An HPLC system equipped with a PDA detector and a C18 column (250 × 4.6 mm, 5 μm) was used, with the column temperature maintained at 37 °C. The mobile phase was a mixture of methanol and water (50:50, *v*/*v*), the flow rate was 1 mL/min, and the detection wavelength was 284 nm.

### 2.7. AA-Induced Acute Colitis Rats

#### 2.7.1. Animals and Experimental Design

Thirty-five male Sprague–Dawley (SD) rats (220 ± 20 g) were purchased from SPF Biotechnology Co., Ltd. (Beijing, China). After 1 week of adaptation, all rats were randomly divided into 5 groups (*n* = 7 per group): CON group, AA group, HD-SD group, HD-PM group, and HD group (the mass ratio of HD-PVPK30 was 1:9 for HD-SD and HD-PM).

Rats in the CON group and the AA group were administered normal saline by intragastric gavage for consecutive 14 days, while rats in the HD-SD group, the HD-PM group, and the HD group were administered HD-SD, HD-PM, and HD (containing 40 mg/kg of HD, suspended in 0.5% CMC-Na solution) in the same way. On the 8th day, diluted AA (2 mL, 3% *v*/*v*) was inserted into the rat (all the rats except the CON group) anus for 30 s using a PVC cannula with a diameter of 2 mm and slowly injected into the rat anus at a depth of 8 cm [29]. To prevent AA leakage, the animals remained in a head-down position for 2 min. All the rats were treated with the specified drugs 1 h after inducing colitis with AA.

#### 2.7.2. Assessment of the DAI (Disease Activity Index) and Sample Collection

From the 8th day, the body weight, stool consistency, and the presence of blood in the stool of each rat were recorded every day. The DAI score was calculated based on the method of the previous report [30]. On day 15, the rats were anesthetized (by inhalation of 2% isoflurane) and sacrificed by cervical dislocation after the blood was collected. The colon of each rat was removed and measured in length. The colon segment was fixed with 4% paraformaldehyde solution. The fixed colon tissue was embedded in paraffin, cut into 5 μm slices, and stained with hematoxylin and eosin (H&E).

#### 2.7.3. ELISA

The levels of interleukin (IL) such as IL-1β, IL-10, IL-6, and tumor necrosis factor-α (TNF-α) were determined from the serum by enzyme-linked immunosorbent assay (ELISA) kits according to the manufacturer’s instructions (Shanghai Jianglai Biological Technology Co., Ltd., Shanghai, China).

#### 2.7.4. Gut Microbiota Analysis

The intestinal contents from the cecum were collected aseptically and the samples were stored at −80 °C for later examination. Total genome DNA from the samples was extracted using the cetyltrimethylammonium bromide/sodium dodecyl sulfate (CTAB/SDS) method. DNA concentration and purity were monitored on 2% agarose gels. The V4 hypervariable regions of the bacterial 16S rRNA gene were amplified using the polymerase chain reaction (PCR) system (Bio-Rad Laboratories) with primers 515F (5′-GTGCCAGCMGCCGCGGTAA-3′) and 806R (5′-GGACTACHVGGGTWTCTAAT′). The PCR products were purified with the Gene JET Gel Extraction Kit (Thermo Scientific, Waltham, MA, USA). The NEB Next^®^ Ultra™ DNA Library Prep Kit for Illumina (NEB, Ipswich, MA, USA) was used to prepare sequencing libraries. The library’s quality was tested using the Qubit@ 2.0 Fluorometer (Thermo Scientific, Waltham, MA, USA) and the Agilent Bioanalyzer 2100 system. Quantified libraries were pooled and sequenced on Illumina platforms based on effective library concentration and data volume requirements. The gut microbiota’s α and β diversity were determined using QIIME (Version QIIME2-202202) software. The species annotation was performed with the QIIME2 program. The ade4 and ggplot2 packages in R (Version 4.0.3) were used to display the principal coordinate analysis (PCoA). Non-metric multi-dimensional scaling (NMDS) analysis was carried out using R software, namely the ade4 and ggplot2 packages.

### 2.8. Statistical Analysis

Pharmacokinetic parameters in vivo were analyzed using DAS 2.0 and SPSS 23.0 statistical software. One-way ANOVA was performed using SPSS 23.0, followed by Duncan’s multiple. *p* < 0.05 was considered statistically significant and *p* < 0.01 was considered to be extremely significant.

## 3. Results

### 3.1. The Characterization of HD-SD

#### 3.1.1. PXRD Analysis

**Figure 1:**
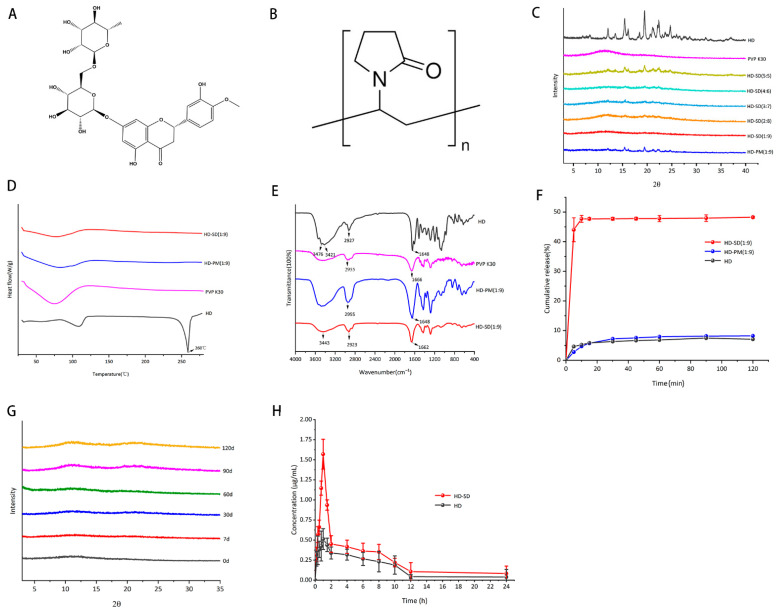
PXRD, DSC, FT-IR, stability and in vivo/in vitro release analysis of HD-SD. (**A**) Chemical structure of HD. (**B**) Chemical structure of PVPK30. (**C**) PXRD patterns of different samples. (**D**) DSC of different samples. (**E**) FT-IR spectra of different samples. (**F**) Dissolution diagram in vitro. (**G**) The PXRD patterns of HD-SD (1:9) at different times. (**H**) Blood concentration–time curves of HD and HD-SD (*n* = 6).

The PXRD results of pure HD, PVPK30, SD, and PM are shown in Figure 1C. The characteristic diffraction peaks of pure HD were 12.03°, 15.42°, 19.46°, 21.09°, 22.34°, and 24.67°, which indicated its crystal form. Furthermore, characteristic diffraction peaks of HD were still present in the HD-SD (2:8), HD-SD (3:7), HD-SD (4:6), HD-SD (5:5), and HD-PM (1:9), while they were absent in the HD-SD (1:9). It was demonstrated that HD maintains the amorphous state in HD-SD (1:9), that is, HD-SD was successfully prepared at this ratio.

#### 3.1.2. DSC Analysis

The DSC thermograms of pure HD, PVPK30, HD-SD (1:9), and HD-PM (1:9) are presented in Figure 1D. A characteristic endothermic peak observed at 260 °C for HD corresponds to its melting point, confirming its crystalline nature [22]. The DSC thermogram of HD-SD (1:9) and HD-PM (1:9) displayed the absence of the endothermic peak of HD.

#### 3.1.3. FT-IR Spectrum Analysis

The FTIR spectra of the HD, PVPK30, SD (1:9), and PM (1:9) are shown in Figure 1E. In the spectrum of crystalline HD, two peaks are present at 3476 cm^−1^ and 3421 cm^−1^, which was caused by the stretching vibration of -OH. The stretching vibrations of C-H and C = O are 2929 cm^−1^ and 1648 cm^−1^, respectively. PVPK30 showed two distinct peaks at 2955 cm^−1^ and 1666 cm^−1^, which corresponded to -OH and C = O stretching. In the spectrum of HD-SD (1:9), the -OH peak of the HD became wider; the -OH characteristic peak of PVPK30 changed from 2955 cm^−1^ to 2923 cm^−1^, and the C = O characteristic peak of HD changed from 1648.3 cm^−1^ to 1662 cm^−1^.

### 3.2. Powder Dissolution

Powder dissolution profiles for HD, HD-SD, and HD-PM in phosphate-buffered saline (PBS, pH 6.8) are shown in Figure 1F. It can be found that the cumulative release of HD and HD-PM within 120 min was 7.06% and 8.18%, respectively. The cumulative release of HD-SD was 47.69% within 10 min and was about 48.24% at 120 min, which was 5.9 times that of HD.

### 3.3. Stability of the Samples

As shown in Figure 1G, there were no HD characteristic diffraction peaks in the PXRD curves during the research. HD-SD (1:9) was stored at 40 °C and 75% RH for 120 days. The results indicated that the HD-SD (1:9) remained in an amorphous state and had good physical stability.

### 3.4. Pharmacokinetic Characteristics

The results of pharmacokinetic studies in vivo are shown in Table 1. Compared with HD, the C_max_ and AUC_0–24_ in HD-SD (1:9) were significantly higher than that of HD (*p* < 0.01), which were 2.67 and 1.50 times that of HD, respectively.

### 3.5. HD-SD Enhanced the Amelioration of Ameliorated AA-Induced Acute Colitis

#### 3.5.1. Assessment of DAI and Colonic Damage

**Figure 2:**
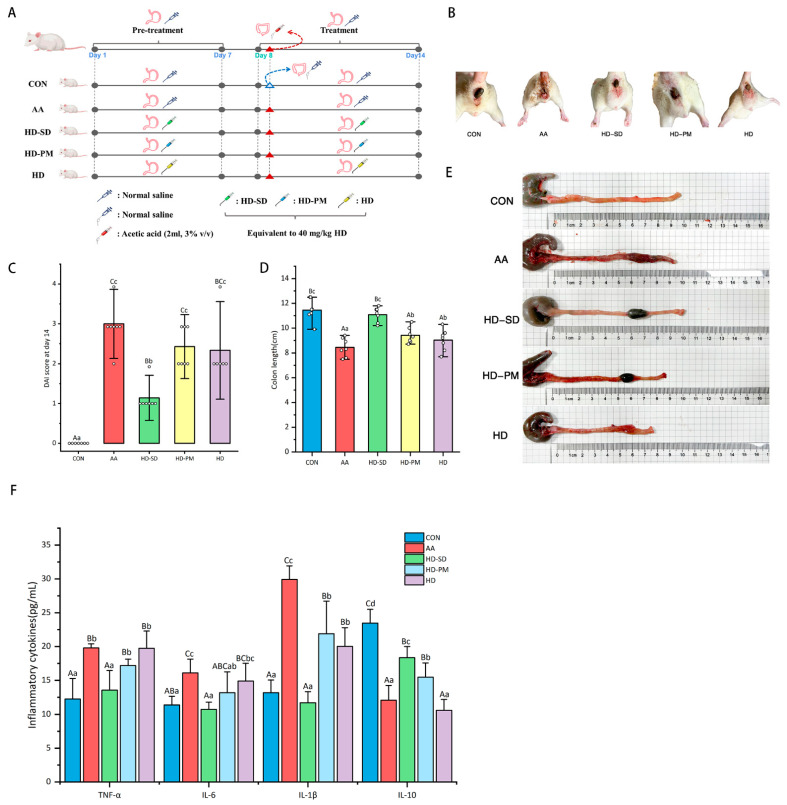
HD-SD attenuated symptoms of AA-induced colitis in rats. (**A**) Diagram of flow for the design of the animal experiment. (**B**) Diarrhea situation of rats. (**C**) Disease activity index (DAI) score of rats on day 14. (**D**) Colon length of the rats. (**E**) Representative images of the colon from rats. (**F**) Changes in the levels of inflammatory cytokines in the serum. Data are presented as mean ± standard deviation (SD) (*n* = 6–7 rats per group). Different lowercase letters indicate significant differences (*p* < 0.05), and different capital letters indicate extremely significant differences (*p* < 0.01). The same letters indicate no significant difference (*p* > 0.05).

**Figure 3:**
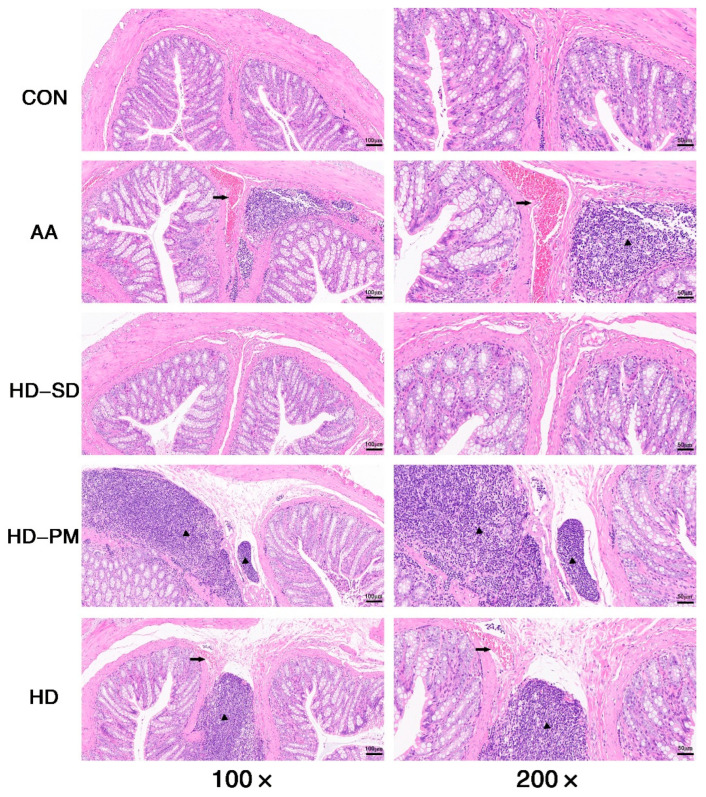
Representative H&E staining of colon tissue sections. The black arrow shows inflammation, and the black triangle shows bleeding.

The animal experimental plan is illustrated in Figure 2A. The fecal status of the rats is shown in Figure 2B. The results showed that the AA group had diarrhea which significantly improved after HD treatment. The severity of the AA-induced colitis was evaluated using the DAI score. At the conclusion of the experiment, the highest DAI scores were observed in the AA group, while the HD-SD group exhibited a significant decrease in these scores (*p* < 0.01). The rats of the HD and HD-PM group exhibited lower DAI scores, and there was no significant difference between these two groups (Figure 2C). The literature reported that AA causes severe inflammation and shortening of the colon [31,32]. As depicted in Figure 2D,E, treatment with HD prevented colon shortening, and the effect of HD-SD was better than PM and HD (*p* < 0.01). Additionally, the colon in the AA group displayed pronounced dark red swelling and the HD-SD group reversed these effects (Figure 2E). In addition, Figure 3 displays the pathological characteristics of the colon slice as assessed by H&E staining; the colon of the AA group exhibited various inflammatory features such as evident bleeding, extensive infiltration of inflammatory cells, and goblet cell depletion. In contrast, HD-SD, HD-PM, and HD supplementation alleviated AA-induced histopathological alterations of the colon.

#### 3.5.2. Examination of Anti-Inflammatory Effects

The effects of HD-SD on TNF-α, IL-6, IL-1β, and IL-10 levels in the serum after AA administration were evaluated. Compared with the CON group, the levels of TNF-α, IL-6, and IL-1β in the AA group were significantly elevated, while the level of IL-10 was significantly reduced (Figure 2F) (*p* < 0.01). In contrast, compared to the AA group, the treatment with HD-SD significantly reduced AA-induced TNF-α, IL-6, and IL-1β production, and significantly enhanced the secretion of the anti-inflammatory cytokine IL-10 (Figure 2F) (*p* < 0.01). Through careful observation, we found that the levels of TNF-α and IL-1β in the HD-SD group were significantly lower than HD-PM and HD groups (*p* < 0.01). Compared to the HD group, the HD-SD group exhibited significantly lower/higher levels of IL-6/IL-10 (*p* < 0.01). Although a change of 27.88%/73.28% was observed, this difference was not statistically significant. All these results indicated that HD with the ability of inhibiting pro-inflammatory cytokines and the effects of HD-SD was better than the PM and HD.

#### 3.5.3. HD-SD Administration Modulated Gut Dysbiosis in Colitic Rat

**Figure 4:**
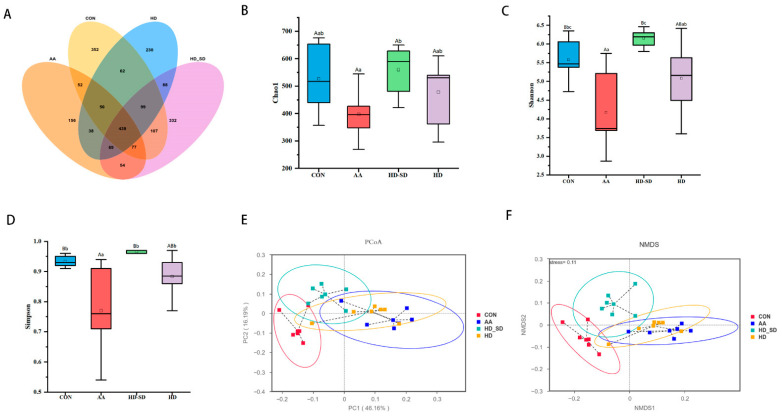
HD-SD improved gut microbiota diversity in AA-treated rats. (**A**) Venn diagram of OTUs. (**B**) Chao1 index. (**C**) Shannon index. (**D**) Simpson index. (**E**) PCoA plot. (**F**) NMDS plot. Data are presented as mean ± SD (*n* = 6 rats per group). Different lowercase letters indicate significant differences (*p* < 0.05), and different capital letters indicate extremely significant differences (*p* < 0.01). The same letters indicate no significant difference (*p* > 0.05).

To investigate the effects of HD-SD on the gut microbiota composition of rats with acetic acid (AA)-induced colitis, 16S rDNA gene amplicons in the intestinal contents of the rats were sequenced. As shown in Figure 4A, the Venn diagram indicated that 2203 operational taxonomic units (OTUs) in all were identified across the four groups of fecal samples. Unique OTUs of the CON, AA, HD-SD, and HD groups were 352, 156, 332, and 230, respectively, indicating greater OUT diversity in the HD-SD group.

Compared to the CON group, the Shannon and Simpson indices were significantly decreased (*p* < 0.01 or *p* < 0.05) in the AA group (Figure 4B–D), showing that AA decreased the gut microbiota’s richness and diversity. There was no statistically significant difference in the Chao1, Shannon, and Simpson indices between the HD-SD and CON groups, suggesting that HD-SD treatment restored the microbiota that resemble that of the CON group. PCoA and NMDS plots were generated to assess the similarities and differences in microbial communities among groups (Figure 4E,F). The results revealed a clear separation in bacterial community composition between the CON and AA groups. Interestingly, the bacterial community of the HD-SD group clustered more closely to the CON group than the AA group, suggesting that HD-SD alleviated the gut microbiota dysbiosis in rats with colitis.

**Figure 5:**
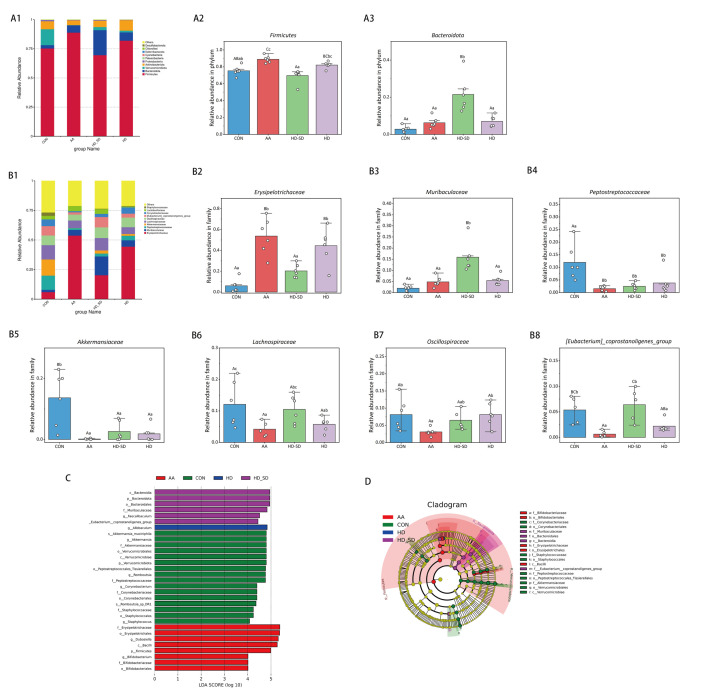
Effect of HD-SD on the gut microbiota compositions and relative abundances. (**A1**) The top 10 microbiota taxa found at the phylum level. (**A2**) *Firmicutes*. (**A3**) *Bacteroidota*. (**B1**) The top 10 microbiota taxa found at the family level. (**B2**) *Erysipelotrichaceae*. (**B3**) *Muribaculaceae*. (**B4**) *Peptostreptococcaceae*. (**B5**) *Akkermansiaceae*. (**B6**) *Lachnospiraceae*. (**B7**) *Oscillospiraceae*. (**B8**) *[Eubacterium]_coprostanoligenes_group*. (**C**) The distribution histogram based on LDA (LDA > 4.0). (**D**) Cladogram of LEfSe. Data are presented as mean ± SD (*n* = 5–6 rats per group). The same letters indicate no significant difference (*p* > 0.05), while different lowercase letters indicate significant differences (*p* < 0.05). Different capital letters indicate extremely significant differences (*p* < 0.01).

The composition of the gut microbiota at the phylum level in intestinal content samples is presented in Figure 5(A1). The relative abundance of *Firmicutes and Verrucomicrobiota* was obviously declined in the AA group (*p* < 0.01), whereas HD-SD supplementation prominently reversed the relative abundance of *Firmicutes* caused by AA treatment (*p* < 0.01) (Figure 5(A2)). Moreover, the relative abundance of *Bacteroidota* was significantly boosted in the HD-SD group (*p* < 0.01) (Figure 5(A3)).

The relative abundance of gut microbiota at the family level in intestinal contents was illustrated in Figure 5(B1). At the family level, the relative abundance of *Erysipelotrichaceae* was significantly elevated in the AA group (*p* < 0.01); these results were consistent with the previous literature [2] (Figure 5(B2)). The administration HD-SD significantly reversed the increase in *Erysipelo-trichaceae*. Moreover, the relative abundances of *Akkermansiaceae* and *Lachnospiraceae* were significantly reduced in the AA group (*p* < 0.01 or *p* < 0.05), whereas HD-SD supplementation prominently reversed the relative abundance of *Lachnospiraceae* caused by AA treatment (*p* < 0.01) (Figure 5(B5,B6)). AA treatment decreased the relative abundance of *Oscillospiraceae* and *[Eubacterium]_coprostanoligenes_group* compared with that of normal saline treatment (*p* < 0.05 or *p* < 0.01), whereas HD-SD supplementation prominently reversed the relative abundance of *[Eubacterium]_coprostanoligenes_group* caused by AA treatment (*p* < 0.01).

We then used LEfSe analysis to identify statistically significant taxa in each experimental group. Figure 5C,D display the outcomes of the LEfSe analysis. Notably, the levels of *Erysipelotrichaceae* and *Erysipelotrichales* were broadened in the AA group. Importantly, *Erysipelotrichaceae* was found to have a positive correlation with ulcerative colitis (UC) disease [33]. The HD-SD group has nine significantly enriched marker species, including *Bacteroidia*, *Bacteroidota*, *Bacteroidales*, *muribaculaceae*, and *oscillospirates*. These bacterial genera are recognized for their capacity to break down complex polysaccharides via fermentation, resulting in the production of short-chain fatty acids [34].

## 4. Discussion

Currently, UC has become a globally prevalent gastrointestinal disorder closely associated with modern dietary patterns, significantly compromising life quality. While current therapeutic approaches often yield suboptimal outcomes, bioactive compounds derived from functional foods emerge as promising candidates for UC prevention and dietary management. Solid dispersion is a kind of solid form that can satisfactorily improve the solubility and the dissolution rates [21]. There are probably two reasons for this: first, the bioactive compounds were uniformly dispersed in the carriers in an amorphous form; second, no energy was required for the bioactive compounds to break the crystal lattice [28]. Polyvinylpyrrolidone K30 (PVPK30) is a commonly used carrier which is a hydrophilic polymer composed of N-vinylpyrrolidone monomers. The molecular weight of PVP ranges from approximately 2.5 to 3000 kDa, with “K” values ranging from 12 to 120, depending on the length of the polymer chain [35]. PVP K30, a hydrophilic and biocompatible polymer, demonstrates low toxicity and dissolves readily in a range of polar solvents. It is widely utilized in diverse drug delivery applications such as oral, topical, and ocular formulations [36]. Additionally, PVP K30 is widely used in the cosmetics, food, and biomedical fields [21]. The novel curcumin analog C086 solid dispersion (C086-SD) was prepared using PVP K30 as a carrier, which had a cumulative dissolution of 100% within 20 min and an oral bioavailability of 28 times that of C086 [37].

The crystalline state of a drug is crucial for the formation of amorphous solid dispersions. PXRD can detect the crystallinity of drugs [38]. It has been recognized that no crystalline diffraction peaks of HD were observed in the HD-SD 1:9 system, indicating the effective production of an amorphous solid dispersion. DSC can analyze the thermal properties of drugs such as melting point, boiling point, and crystallinity [39]. From the DSC analysis, a significant endothermic peak was observed for HD at 260 °C which was consistent with the literature [22]. A broad endotherm ranging from roughly 50 °C to 100 °C was observed in the thermogram of the PVP, showing water loss due to the extremely hygroscopic properties of PVP polymers [40]. No endothermic peak of HD was observed in the PM, possibly due to a higher proportion of polymer. And with the temperature increased, PVPK30 melted before HD, obscuring HD’s melting peak [28,41]. The absence of an endothermic peak of HD in HD-SD indicates the amorphous state.

The low solubility of HD leads to low oral bioavailability [42]. The dissolution curve showed that the cumulative release of HD-SD (1:9) was higher than that of HD and HD-PM (1:9), indicating that the amorphous state of HD could improve the dissolution performance of HD. The enhancement of release of HD-SD in vivo and in vitro is facilitated by the presence of the hydrophilic PVP carrier, which improves the wetting properties of the amorphous drug. PVPK30 promotes fast release in addition to improving the solubility of poorly soluble medications [43]. Importantly, polymer adsorption onto the surface of crystalline medicines may be encouraged by interactions between the drug and carrier, inhibit crystal growth and disperse them into an amorphous or metastable microcrystalline state within the water-soluble carrier, and thereby enhance the solubility [44]. Additionally, mechanical milling induces changes in particle morphology and distribution, promotes favorable surface effects, size effects, and quantum effects, thereby enhancing drug activity and adsorption properties, and consequently improving the dissolution rate and bioavailability [38]. Furthermore, pharmacokinetic results indicate that the C_max_ and AUC_0–24_ of HD-SD are 2.67 and 1.50 times higher than HD, respectively. This improvement was attributed to the close relationship between drug solubility and bioavailability [45].

The model of colitis in rats was established using the rectal administration of acetic acid, which was reported in the previous literature [46,47]. DAI is a comprehensive assessment method used to measure disease activity, particularly in IBD. The DAI score combines measurements of weight loss, stool consistency, and bleeding, calculating these indicators to assess the severity of the disease. This method plays a crucial role in the diagnosis and monitoring of inflammatory diseases such as IBD [48]. Our results indicated that the AA group had the highest DAI score, while the HD-SD group had the lowest DAI score. Additionally, a significant characteristic of UC is the marked shortening of the colon, which is consistent with our experimental results [49]. As shown in the figure, the AA-treated colons were notably shortened and exhibited congestion, whereas the HD-SD intervention significantly improved these conditions. Histopathological examination is a critical tool in pathology research, allowing for the observation of inflammatory infiltration, ulcer formation, and mucosal hemorrhage in colitis [50]. Histopathological results revealed significant hemorrhage and inflammatory infiltration in the AA group, while the HD-SD intervention ameliorated these symptoms. Furthermore, this model induces the excessive production of pro-inflammatory cytokines such as TNF-α, IL-1β, and IL-6, and is characterized by congestion, ulceration, and a reduction in goblet cells, leading to persistent inflammation [29]. This is consistent with our experimental findings. Overall, every treatment in this study improved colitis; however, the HD-SD intervention showed superior efficacy compared to the other treatment groups. This may be closely related to the water solubility of hesperidin. As evidenced by research, optimized hesperidin magnesium improved the water solubility of pure hesperidin, resulting in better therapeutic effects on UC compared to pure hesperidin [51].

Imbalance in the gut microbiota is closely associated with impaired intestinal barrier function and inflammatory responses. The balanced state of gut microbial communities is crucial for maintaining the stability of the intestinal ecosystem [52]. To investigate the effects of HD-SD on the gut microbiota in rats with colitis, this study analyzed the alpha and beta diversity, as well as the composition of gut communities, based on 16S rDNA high-throughput sequencing. Research indicates that UC patients’ gut microbiota diversity is significantly reduced than that of healthy individuals [34]. The α-diversity and β-diversity analysis indicated that the diversity and compositional structure of intestinal flora in the CON group were remarkably different from those in the AA group, and the CON group showed more similarity with the HD-SD groups. These results showed that HD-SD could recover the variety and composition of the gut microbial community in AA-treated rats. Furthermore, at the phylum level, the relative abundances of *Firmicutes* were significantly decreased in the AA group, and the relative abundances of *Bacteroidota* were significantly increased in the HD-SD group. The two most abundant phyla in the intestines of both humans and rats are *Bacteroidetes and Firmicutes*, relying mainly on carbohydrate-active enzymes for the utilization of dietary polysaccharides [53]. They can be major degraders of complex carbohydrates. At the family level, the relative abundances of *Akkermansiaceae* and *Lacnnospiraceae* were significantly reduced in the AA group, which was in line with what was found in earlier research. *Lachnospiraceae* is consistently decreased in patients with IBD, as has been demonstrated. It has been reported that *Lachnospiraceae* exhibits strong capability in polysaccharide degradation and production of SCFAs, potentially contributing to the breakdown of ASPP [54]. *Lachnospiraceae* is a major generator of short-chain fatty acids in the gut, which is essential for intestinal health [55]. *Akkermansiaceae*, a microbe that plays a positive role in maintaining intestinal barrier homeostasis, can increase the levels of mucin, thereby restoring intestinal barrier function and reducing colonic inflammation [2,6]. Compared to the AA group, HD-SD intervention enriched *Lachnospiraceae* and *Akkermansiaceae*. Additionally, an increase in *Erysipelotrichaceae* is frequently observed in animals with UC [2], in agreement with the results obtained herein. In addition, HD-SD supplementation significantly reversed the decrease in the abundance of *[Eubacterium]_coprostanoligenes_group* caused by AA treatment. One study found that *[Eubacterium]_coprostanoligenes_group* can strengthen the integrity of the intestinal mucus barrier by promoting mucin secretion by goblet cells, which helps to resist microbial invasion and subsequently reduce inflammation [56].

The LEfSe results indicated that the marker species included *Erysipelotrichaceae* and *Erysipelotrichales* which were significantly enriched in the AA group. Feng et al. showed that the polysaccharides can ameliorate the *Erysipelatoclostridium* elevation caused by DSS [57], which is consistent with the results of this study. In addition, AA treatment notably decreased the relative abundance of *Oscillospiraceae*. *Oscillospiraceae* produces SCFAs, such as butyrate, which play an important role in gut health. Butyrate is not only an energy source, but also has anti-inflammatory properties. However, HD-SD supplementation considerably alleviated the aforementioned alterations generated by the AA treatment. *Bacteroidetes*, recognized as beneficial gut microbes, play a critical role in preventing intestinal diseases such as inflammation and maintaining gut health [34]. The HD-SD group showed significant enrichment of *Bacteroidota*, suggested that HD-SD intervention improves the gut microbiota structure. Collectively, the integration of food-derived HD with PVPK30 presents a promising treatment strategy for UC management, exemplifying the translational potential of food-grade delivery systems in preventive health applications.

## 5. Conclusions

HD-SD were successfully synthesized using PVPK30 as the carrier in this study. Compared with pure HD, HD-SD showed a higher cumulative dissolution rate and higher bioavailability. In addition, HD-SD has advantages in effectively alleviating bloody diarrhea, colonic tissue damage, and colonic inflammation in AA-induced colitis rats. HD-SD was able to correct the dysregulated gut microbiota structure through increasing the relative abundance of beneficial bacteria and decreasing that of harmful bacteria. Therefore, the results suggested that HD-SD has potential as a functional food or as a novel therapeutic strategy for UC.

## Figures and Tables

**Table 1:** Pharmacokinetic parameters of HD and HD-SD (1:9) (*n* = 6).

Items	Group	
	HD	HD-SD
T_max(0–24)_ (h)	1.15 ± 0.34	0.96 ± 0.10
C_max(0–24)_ (μg/mL)	0.61 ± 0.14	1.63 ± 0.17 **
t_1/2(0–24)_ (h)	5.35 ±1.69	4.30 ± 1.03
AUC_0–24_ (μg·h/mL)	4.05 ± 0.55	6.06 ±0.81 **

Results are presented as “mean ± standard deviation.” Compared with HD, ** *p* < 0.01 indicates a significant difference.

## Data Availability

The original contributions presented in the study are included in the article. Further inquiries can be directed to the corresponding authors.

## Abbreviations

HD, Hesperidin; PVPK30, polyvinylpyrrolidone K30; SD, solid dispersion; IBD, inflammatory bowel disease; CD, Crohn’s disease; UC, ulcerative colitis; AA, acetic acid; API, active pharmaceutical ingredient; HD-SD, hesperidin solid dispersion; PXRD, Powder X-ray Diffraction; DSC, Differential Scanning Calorimetry; FTIR, Fourier Transform Infrared Spectroscopy; DAI, disease activity index; H&E, hematoxylin and eosin; PCoA, principal coordinate analysis; NMDS, non-metric multi-dimensional scaling.
